# Comparison of Vacuum-Assisted Closure Therapy Versus Conventional Treatment for Post-sternotomy Mediastinitis: A Retrospective Study From a Tertiary Care Centre

**DOI:** 10.7759/cureus.84143

**Published:** 2025-05-15

**Authors:** Md Intekhab Akram, Faiz Khan Yusufi, Syed Shamayal Rabbani, Aamir Mohammad, Azam Haseen

**Affiliations:** 1 Cardiothoracic Surgery, Jawaharlal Nehru Medical College, Aligarh, IND; 2 Surgery, Jawaharlal Nehru Medical College, Aligarh, IND; 3 Cardiothoracic and Vascular Surgery, Jawaharlal Nehru Medical College Hospital, Aligarh, IND

**Keywords:** coronary artery bypass grafting (cabg), mediastinitis, sternotomy, vacuum-assisted closure, wound

## Abstract

Background: A median sternotomy is widely utilised as the standard approach for open cardiac surgeries to manage heart conditions like heart failure, valve disorders, irregular heart rhythms as part of arrhythmia surgery, aneurysms, and coronary artery disease, with coronary artery bypass grafting (CABG) being a common procedure. Despite the application of proper aseptic techniques, perioperative antibiotics, and meticulous wound care, surgical site infections, such as deep sternal wound infections (DSWI) or mediastinitis, can still develop.

Methodology: Between October 2016 and December 2023, 1,542 osteotomies were performed on patients undergoing cardiac surgeries in the Department of Cardiothoracic and Vascular Surgery, Jawaharlal Nehru Medical College, Aligarh. Thirty-six patients (2.34%) were diagnosed with mediastinitis post-cardiac surgery. These patients were analysed retrospectively for demographics and clinical information comprising of medical history, vital signs, findings from a comprehensive clinical examination, laboratory results, comorbidities, prior surgeries, duration of hospital stay, and the indication for the current procedure, and two groups were formed: Group 1 (n = 20), who were treated with vacuum-assisted closure (VAC) therapy, and Group 2 (n = 16), who received conventional treatment.

Results: The mean age of patients in the vacuum-assisted closure group was 38.9 ± 4.5 years, while the conventional treatment group had a mean age of 43.6 ± 5.0 years; other baseline characteristics were comparable in both groups. The average length of hospital stay after VAC therapy was 13 ± 2.5 days, with 16 patients requiring intensive care unit admission for more than three days. The conventional treatment group had an average stay of 23 ± 3.2 days, with eight patients needing ICU care for over three days. The duration of treatment, calculated from the time of mediastinitis diagnosis, was 16 days in the VAC group compared to 22 days in the conventional treatment group. The mortality rate in our study was 5% in the VAC therapy group and 6.25% in the conventional treatment group. Both patients who died did so due to multiorgan failure resulting from severe sepsis.

Conclusion: VAC therapy can be considered a good alternative to more aggressive surgery that might not be suitable for some patients during certain times of their treatment course. It can also be used as a bridge to reconstructive surgery by improving the wound situation and the patient's general condition. This will improve the clinical outcome and patient satisfaction. VAC therapy for poststernotomy mediastinitis is an effective treatment, which speeds up wound granulation tissue formation and accelerates wound closure with decreased need for more aggressive reconstructive surgery. Moreover, accelerated wound closure may be associated with fewer complications and reduced expenses.

## Introduction

Open-heart surgery is performed to manage a wide range of cardiac conditions, including heart failure, valve disorders, irregular heart rhythms (as addressed in arrhythmia surgery), aneurysms, and coronary artery disease, with coronary artery bypass grafting (CABG) being among the most commonly performed procedures. In addition to these acquired conditions, a significant portion of open-heart surgeries involve the correction of congenital heart defects. Common congenital cardiac surgeries include atrial and ventricular septal defect closures, tetralogy of Fallot repair, corrected transposition of the great arteries, and procedures addressing complex anomalies such as hypoplastic left heart syndrome. These surgeries are critical in improving survival and quality of life in pediatric and adult congenital heart disease patients. Including data on congenital cardiac surgery allows for a comprehensive understanding of the scope and impact of open-heart surgical interventions. 

Almost 400,000 CABG surgeries are performed each year, making it the most commonly performed major surgical procedure for cardiac surgeries [1]. A median sternotomy is widely utilised as the standard approach for these cardiac surgeries. Despite the application of proper aseptic techniques, perioperative antibiotics, and meticulous wound care, surgical site infections, such as deep sternal wound infections (DSWIs) or mediastinitis, can still develop. The incidence of mediastinitis ranges from 0.8% to 5%, but it is associated with severe complications. Mortality rates in adult cardiac surgical patients have been reported to range from 19% to 29% in various studies [1-5]. Mediastinitis also adds to the patient's financial burden by prolonging hospital stays and necessitating additional treatment costs. The most frequently identified pathogens in DSWIs are *Staphylococcus aureus* and coagulase-negative staphylococci [6,7]. The treatment of mediastinitis varies widely, ranging from straightforward antibiotic therapy to complex surgical interventions, such as omental or myocutaneous flap reconstruction [8,9]. Traditional treatment for mediastinitis typically involves surgical wound debridement, repeated wound irrigation, and sternal reconstruction combined with antibiotics and disinfectants. However, these conventional methods are often linked to high rates of mortality and morbidity, prolonged hospital stays, and a significant risk of recurrence. 

Over time, alternative therapeutic approaches have been developed. Vacuum-assisted closure (VAC), which works on the principle of negative-pressure wound therapy (NPWT), is a noteworthy advancement in treatment. This technique involves applying controlled suction through a porous dressing to facilitate wound granulation and closure. VAC therapy allows continuous open drainage, effectively absorbing exudate, stabilising the mediastinal cavity, and isolating the wound. In addition, it promotes the proliferation of granulation tissue and enhances blood flow to the surrounding tissues [10]. VAC therapy also helps to reduce excess fluid and oedema, minimises bacterial colonisation at the wound site, and decreases the frequency of required dressing changes [11]. Promising results have been reported in multiple studies regarding the use of VAC therapy for the management of post-cardiac surgery mediastinitis. VAC therapy has been shown to reduce mortality, lower the risk of treatment failure, shorten hospital stays, and even be applicable in outpatient settings [1,12,13]. VAC therapy is also linked to a lower incidence of postoperative complications that necessitate re-operation after definitive wound closure [14].

This study is designed to evaluate and compare the patient characteristics, clinical outcomes, and survival rates of individuals treated with either VAC therapy or conventional methods for mediastinitis following cardiac surgery.

## Materials and methods

Between October 2016 and December 2023, 1,542 osteotomies were performed on patients undergoing various cardiac surgeries in the Department of Cardiothoracic and Vascular Surgery (CVTS), Jawaharlal Nehru Medical College, Aligarh. A retrospective analysis was conducted to evaluate the incidence of mediastinitis in these cases. The study received ethical approval from the Institutional Ethics Committee of Jawaharlal Nehru Medical College, Aligarh Muslim University (AMU) (approval no. IECJNMC/1440). Mediastinitis was diagnosed in 36 patients (2.34%) following cardiac procedures, based on criteria outlined by the Centres for Disease Control and Prevention (CDC), USA [15]. 

The diagnosis was made if one of the following was present: (1) an organism was isolated from the culture of mediastinal tissue or fluid; (2) evidence of mediastinitis was seen during the operation; or (3) one of the following conditions, chest pain, sternal instability (detachment), or fever (>38°C) was present, and there was either purulent discharge from the mediastinum or an organism isolated from blood culture or drainage culture from the mediastinal area [15]. El-Oakley and Wright's classification system was used to classify the mediastinitis wound (9). 

The inclusion criteria are all patients above the age of one month, diagnosed with mediastinitis in accordance with the CDC criteria post sternotomy for cardiac surgery. The exclusion criteria are neonates undergoing cardiac surgery and patients performing cardiac procedures via approaches other than sternotomy (e.g., minimally invasive techniques).

Patient demographics and clinical information were extracted from their case files, which comprised medical history, vital signs, findings from a comprehensive clinical examination, laboratory results, comorbidities, prior surgeries, duration of hospital stay, and the indication for the current procedure. A retrospective analysis of patient characteristics was conducted, and based on the treatment method received, two groups were formed: Group 1 (n = 20), who were treated with VAC therapy, and Group 2 (n = 16), who received conventional treatment.

Conventional treatment

The Department of CTVS was established at our institution in October 2016, and VAC therapy for managing post-sternotomy mediastinitis was introduced in 2019. During the initial period, all patients who developed post-sternotomy mediastinitis (n = 16) were treated using conventional methods. This approach included debridement to remove necrotic tissue and fibrin, repeated wound irrigation, sternal re-wiring using the Robicsek technique, and various sternal reconstruction procedures performed under antibiotics and disinfectants. Substernal tissue cultures were obtained to identify the infectious pathogens and assess antibiotic resistance patterns. After diagnosing mediastinitis, patients in both treatment groups received prophylactic antibiotics, specifically meropenem and vancomycin, administered in weight-adjusted doses. These antibiotics were continued in the conventional treatment group until culture results were available.

VAC group

After December 2019, all patients with post-sternotomy mediastinitis at our centre (n = 20) were managed using VAC therapy, implemented with the DMP VEL NeXT™ NPWT system. This technique involves applying a foam dressing over the wound, which is then sealed with a thin film layer (400-600 microns thick). The film has an opening that connects a rubber tube to a vacuum pump. The foam and wound are secured with adhesive tape to create an airtight environment. The vacuum pump applies a continuous negative pressure of 75 mm Hg, effectively removing fluids and infection from the wound while aiding in approximating wound edges. Once patients achieve vital stability, they are discharged with the foam dressing in place and scheduled for follow-up visits in the outpatient department every four to five days. VAC was applied for the duration of two to three weeks. During these visits, wound debridement is performed, and dressings are changed. Following the initiation of VAC therapy, antibiotic treatment is downgraded to broad-spectrum options such as amoxicillin and clindamycin, reducing antibiotic use and lowering the risk of antimicrobial resistance.

Statistical analysis

The data collected were entered, processed, and analysed using IBM SPSS Statistics for Windows, Version 26.0 (released 2019, IBM Corp., Armonk, NY). Differences between two or more groups of qualitative variables were evaluated using the Chi-square test (χ2). An independent sample t-test compared two normally distributed (parametric) data groups. Statistical significance was considered for a p-value of less than 0.05. The test statistics were used to present the results of the tests.

## Results

**Figure 1:**
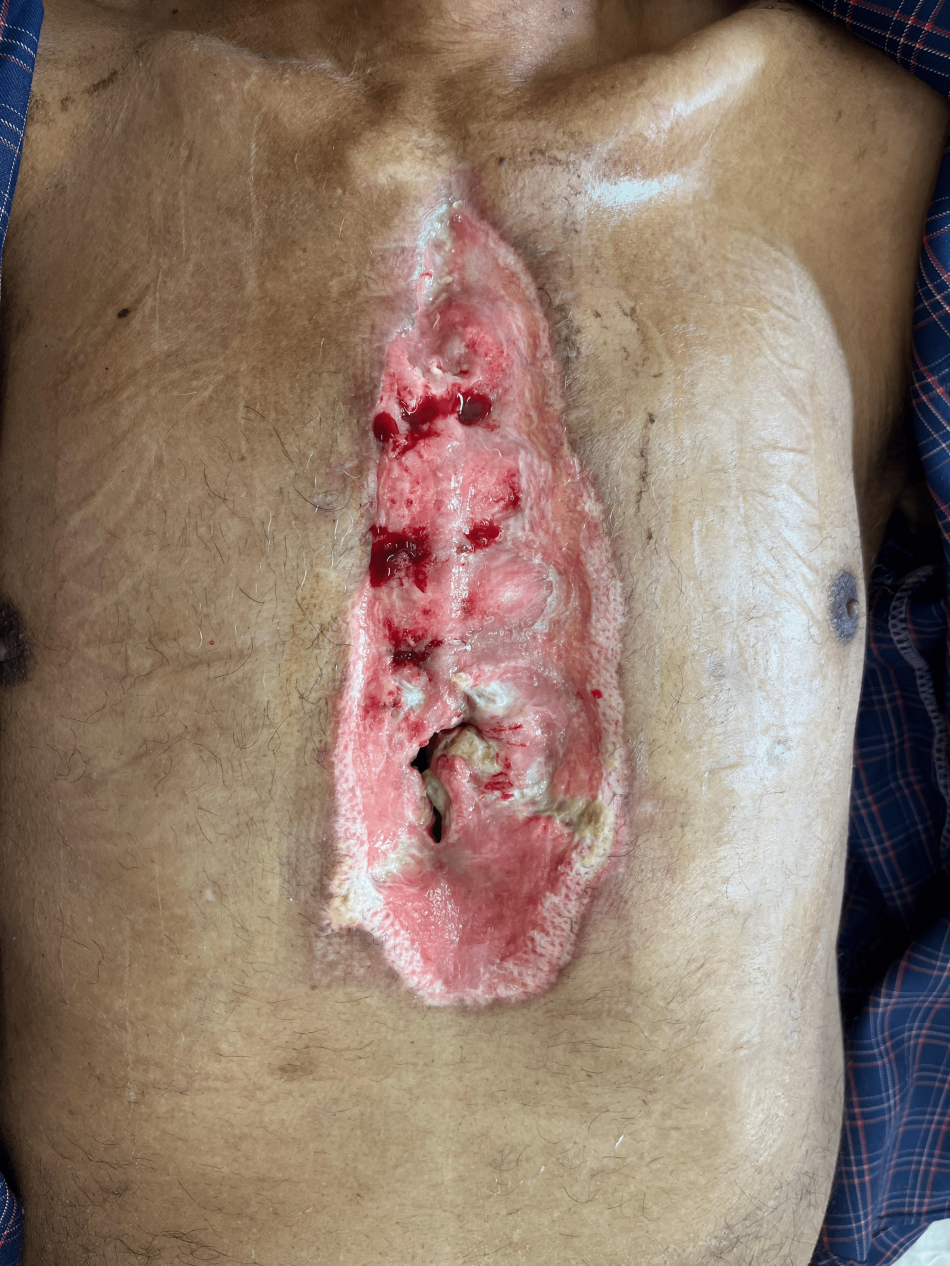
Pre-VAC wound status VAC: vacuum-assisted closure

**Figure 2:**
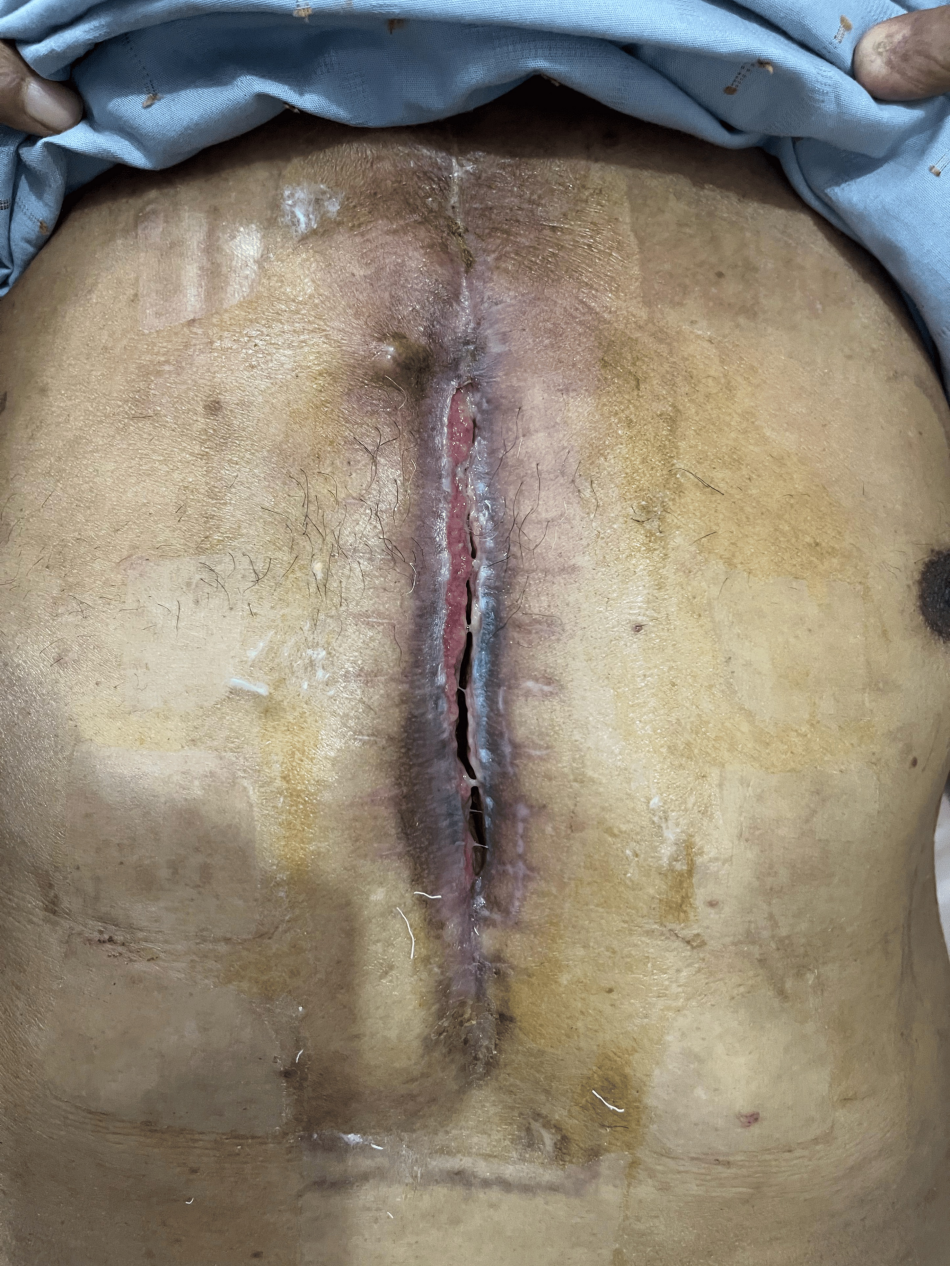
Post-VAC therapy wound status VAC: vacuum-assisted closure

A total of 36 patients (20 VAC, 16 conventional) were included. Gender distribution was similar (p = 0.936, χ² = 0.007, df = 1, effect size = 0.013). The mean age was 38.9 ± 4.6 years in the VAC group and 43.6 ± 7.3 years in the conventional group. Patients aged >60 were comparable (12 vs. 10; p = 0.878, χ² = 0.023, effect size = 0.025). Surgical procedures were similarly distributed, including isolated CABG (p = 0.593, χ² = 0.286), valve replacements (p = 0.549, χ² = 0.360), CABG + additional procedures (p = 0.829, χ² = 0.046), congenital surgeries (p = 0.925, χ² = 0.009), valve repairs (p = 0.364, χ² = 0.823), and other procedures (p = 0.418, χ² = 0.655). All had negligible effect sizes. Comorbidities showed no significant differences: diabetes mellitus (p = 1.000, χ² = 0.001), BMI >30 (p = 0.686, χ² = 0.164), BMI <18.5 (p = 1.000, χ² = 0.001), COPD (p = 0.925, χ² = 0.009), hypertension (p = 0.393, χ² = 0.728), anemia (p = 0.720, χ² = 0.129), smoking (p = 0.650, χ² = 0.206), creatinine >1.3 mg/dL (p = 0.871, χ² = 0.026), emergency surgery (p = 0.406, χ² = 0.689), redo surgery (p = 0.346, χ² = 0.887), re-exploration (p = 0.925, χ² = 0.009), LVEF <0.30 (p = 0.549, χ² = 0.360), NYHA class III-IV (p = 0.418, χ² = 0.655), and LCOS (p = 0.257, χ² = 1.286). Effect sizes across all variables were minimal. Figure 1 and Figure 2 show the wound status pre and post VAC therapy, respectively.

Overall, the two groups were well-matched across demographic and clinical parameters, ensuring comparability for outcome analysis (Table 1).

**Table 1:** Demographic data and patient characteristics CABG: coronary artery bypass graft, COPD: chronic obstructive pulmonary disease, LVEF: left ventricular ejection fraction, NYHA: New York Heart Association, LCOS: low cardiac output syndrome, BMI: body mass index

Variable	VAC therapy	Conventional treatment	P-value	Chi-square statistics	Degree of freedom	Effect size
Number of patients	20	16				
Male	14	11	0.936	0.007	1	0.013
Female	6	5				
Age	38.9 ± 4.6	43.6 ± 7.3				
Age range	0.5-88.6	0.7-85.7				
Age >60 years	12	10	0.878	0.023	1	0.025
Surgical procedure						
Isolated CABG (n = 289)	7	7	0.593	0.286	1	0.089
Isolated valve replacement (n = 409)	4	2	0.549	0.360	1	0.100
CABG + Additional procedure (n = 118)	3	2	0.829	0.046	1	0.036
Congenital cardiac surgery (n = 587)	4	3	0.925	0.009	1	0.016
Valve repairs (n = 35)	1	-	0.364	0.823	1	0.151
Other cardiac procedures (n = 104)	1	2	0.418	655	1	0.135
Comorbidity						
Diabetes mellitus	10	8	1.000	0.001	1	0.004
BMI > 30	2	1	0.686	0.164	1	0.067
BMI < 18.5	5	4	1.000	0.001	1	0.004
COPD	4	3	0.925	0.009	1	0.016
Hypertension	14	9	0.393	0.728	1	0.142
Pre-operative anemia	4	4	0.720	0.129	1	0.060
Smoking (current/past)	9	6	0.650	0.206	1	0.076
S. creatinine > 1.3 mg/dl	1	1	0.871	0.026	1	0.027
Emergency surgery	3	1	0.406	0.689	1	0.138
Redo surgery	5	2	0.346	0.887	1	0.157
Re exploration	4	3	0.925	0.009	1	0.016
LVEF <0.30	4	2	0.549	0.360	1	0.100
NYHA class III-IV	15	10	0.418	0.655	1	0.135
LCOS	10	5	0.257	1.286	1	0.189

Hypertension was the most common comorbidity, affecting 23 patients, followed by diabetes in 18 patients. Only three patients were obese (BMI >30), while nine patients had a low BMI (BMI <18.5). This is likely due to the low socioeconomic status of the population. The two groups had no significant differences regarding these demographic factors or comorbid conditions. In our study, the average length of hospital stay after VAC therapy was 13 ± 2.5 days, with 16 patients requiring ICU admission for more than 3 days. The organism isolated in each case is given in Table 2.

**Table 2:** Culture-verified deep sternal wound infection pathogens VAC: vacuum-assisted closure

Microbial organism	VAC therapy (n = 20)	Conventional treatment (n = 16)
Staphylococcus aureus	3	3
Klebsiella pneumoniae	2	1
Staphylococcus epidermidis	1	1
Escherichia coli	1	1
Streptococcus pneumoniae	1	1
Pseudomonas aeruginosa	4	3
Acinetobacter baumannii	2	1
No growth	6	5

The conventional treatment group had an average stay of 23 ± 3.2 days, with eight patients needing ICU care for over three days. The duration of treatment, calculated from the time of mediastinitis diagnosis, was 16 days in the VAC group compared to 22 days in the conventional treatment group. Table 3 illustrates a comparison of outcomes between the VAC therapy and conventional treatment groups. A significantly higher proportion of patients in the conventional group required ICU stay for more than three days (χ² = 8.73, df = 1, p = 0.0031, Cramér’s V = 0.49). The mortality rate in our study was 5% in the VAC therapy group and 6.25% in the conventional treatment group, with no statistically significant difference (χ² = 0.0022, df = 1, p = 0.96, Cramér’s V = 0.01). Although reinfection occurred only in the conventional group (12.5%), the difference was not statistically significant (χ² = 2.35, df = 1, p = 0.125, Cramér’s V = 0.28). These findings suggest better short-term outcomes in terms of ICU stay and reinfection trends in the VAC group, though overall mortality remained comparable.

**Table 3:** Conventional treatment group versus vacuum-assisted closure treatment group regarding the mortality rate, reinfection rate, in-hospital and ICU stay, and duration of therapy ICU: intensive care unit

Variable	VAC therapy	Conventional treatment	p-value	Chi-square test	Degree of freedom	Effect size
ICU stay >3 days	8	16	0.058	8.73	1	0.49
Hospital stay	13 days ± 2.5 (11-18)	23 ± 3.2 (19-29)				
Mortality	1	1	0.871	0.002	1	0.01
Duration of therapy	16 ± 3.5 (12-29)	22 ± 4.0 (16-35)				
Reinfection rate	0	2	0.125	2.35	1	0.28

Both patients who died did so due to multiorgan failure resulting from severe sepsis. The reinfection rate was 12.5% in the conventional group. Treatment failure occurred in 40% (n = 8) of the VAC therapy group and 31.25% (n = 5) of the conventional treatment group. These patients were classified as El Oakley class type IV A and type IV B (Table 4).

**Table 4:** El Oakley and Wright's classification of mediastinitis

El Oakley and Wright type	VAC therapy (n = 20)	Conventional treatment (n = 16)
I	1	1
II	1	1
IIIA	8	7
IIIB	2	2
IV A	5	3
IV B	3	2
V	--	--

No significant differences were observed between the two groups regarding other risk factors, including diabetes, hypertension, COPD, renal failure, low LVEF, emergency surgery, redo surgery, re-exploration, or low cardiac output state (LCOS). There was no association found with preoperative anaemia or smoking (current or past).

## Discussion

Mediastinitis is a severe complication that may arise following cardiac surgery performed via a median sternotomy approach, with incidence rates ranging from 0.8% to 6% and mortality rates between 10% and 25% reported in different studies [1,16,17]. In our study, mediastinitis was diagnosed based on the CDC criteria [15], revealing an incidence rate of 2.34% among 1,542 osteotomies performed.

Initially, mediastinitis management involved surgical debridement to remove necrotic tissue and any exposed or infected foreign material, wound irrigation (including multiple open dressing changes), antibiotic therapy, and sternal re-wiring [18]. However, this conventional approach is linked to high mortality rates and several disadvantages. The destabilisation of the thoracic cage or sternum often necessitates mechanical ventilation and prolonged immobilisation, which can lead to additional complications such as pneumonia, deep vein thrombosis, and muscle weakness [19]. The extended hospital stays also contribute to a higher financial burden on patients and significantly affect their psychological well-being [20,21]. 

Mediastinitis can be managed with an alternative approach involving sternal debridement followed by reconstruction using vascularised tissue flaps, such as omentum or pectoral muscle flaps. This method offers rapid and reliable eradication of deep infections with low mortality rates, although it may be associated with morbidity related to the flaps [8,22]. In the late 1990s, VAC therapy was introduced as a treatment option for post-sternotomy mediastinitis [10,23]. This NPWT promotes the proliferation of granulation tissue, reduces oedema and interstitial fluid, and helps limit bacterial colonisation [20,24].

VAC is a recent technical innovation in wound care with a growing number of applications. This wound-healing system was developed in the U.S. by Argenta and Morykwas in the mid-1990s. During approximately the same time period, a similar system using sub-atmospheric pressure in wound healing was evaluated by Fleischmann in Germany. VAC therapy has been available in North America since 1995, and the system was introduced in Europe in 1997. This wound-healing technique is based on the application of local negative pressure to a wound. This is achieved by placing polyurethane foam with an open pore structure of 400-600 μm in the wound. One end of a non-collapsible tube is then connected to the foam, and the other end is connected to a vacuum source in a closed system via a fluid container. The foam and the entire wound are covered with an adhesive drape, thus ensuring an air-tight system. Finally, a predetermined, continuous or intermittent negative pressure is applied to the wound. The foam dressing collapses on application of the negative pressure and transmits an even distribution of pressure across the wound [25]. VAC therapy has resulted in better outcomes for infection resolution and faster healing times compared to conventional treatments [10,11]. Since December 2019, all patients at our centre with post-sternotomy mediastinitis (n = 20) have been treated with VAC therapy as the primary treatment, with or without sternal re-wiring and skin closure and without the use of tissue flaps. Bacteriological cultures from the patients revealed that *Pseudomonas aeruginosa* was the most common pathogen (n = 7), followed by *Staphylococcus aureus* (n = 6). However, in several studies on mediastinitis, *Staphylococcus aureus* was typically the most commonly isolated pathogen [4,5,6,26,27]. In 11 patients, mediastinal fluid cultures showed no growth, likely due to prior antibiotic treatment.

The mean age of patients in our study was 41.3 ± 4.8 years, which is lower than in other studies, as we included pediatric patients in our analysis [20,27]. No significant differences were observed between the two groups regarding baseline characteristics, such as age, gender, surgical procedure performed, and the presence of comorbidities such as hypertension (HTN), diabetes mellitus (DM), obesity, BMI <18, chronic obstructive pulmonary disease (COPD), pre-operative anaemia, smoking, and renal failure. In addition, there were no differences in factors like emergency surgery, redo surgery, re-exploration, left ventricular ejection fraction (LVEF) <0.3, NYHA class III or IV, or a low cardiac output state. In our study, the mortality rate was 5% (one patient) in the VAC therapy group, while the conventional treatment group had a mortality rate of 6.25% (one patient). Both patients died from septic shock and multiple organ failure. A study by Deniz et al. involving 90 patients reported a higher overall mediastinitis-related mortality rate of 15.6%, with significantly lower mortality in the VAC group compared to the conventional treatment group (8.5% or four patients versus 23.2% or 10 patients; p < 0.05) [19]. A study by Roemer et al. reported an in-hospital mortality rate of 12.4% in the VAC therapy group, which was significantly lower than the conventional treatment group, which had a mortality rate of 41.7% (p = 0.0032) [20]. In our study, the lower mortality rates in both treatment groups compared to those in Roemer's study may be attributed to including pediatric patients in our population, who generally have better healing capabilities and fewer comorbidities compared to adults.

In our study, the average duration of VAC therapy was 16 days (range 12-29), which was significantly shorter than the conventional treatment group's duration of 22 days (range 16-35; p < 0.05). Similar findings have been observed in other studies, such as Farghaly et al., who reported an average VAC therapy duration of 12.7 ± 6.26 days (range 4-27), and Fleck et al., whose study showed a mean ± SD of 11 ± 8 days [23,28]. Simek et al. found the median VAC treatment time to be 9.2 days (range 6-21 days), with a statistically significant reduction in hospital stay length compared to the conventional treatment group (p < 0.05) [29]. By contrast, a study by Sjögren et al. on 101 patients reported the mean duration of VAC therapy as 11.9 ± 9.0 days. However, they found no significant difference in treatment duration between the two groups [30].

In our study, the average length of hospital stay was 13 days (range 11-18) for the VAC therapy group, compared to 23 days (range 19-29) for the conventional treatment group. These findings are consistent with those of Kamel et al., who reported a mean hospital stay of 12.18 ± 1.92 days following VAC therapy. Similarly, Doss and colleagues also observed a shorter length of stay and treatment duration with VAC therapy. The significantly shorter hospital stay in the VAC group than in the conventional treatment group may be attributed to our hospital policies. Once patients receiving VAC therapy become vitally stable, we discharge them with follow-up appointments every four to five days for wound debridement and foam dressing changes. This approach reduces the length of hospital stays, enhances patient comfort and minimises overall treatment costs, which is crucial given that many of our patients come from low socioeconomic backgrounds. In addition, early discharge benefits the hospital by increasing bed turnover, allowing us to offer more patients other potentially life-saving surgeries. 

Limitations of the study

The first limitation of this study is the small sample size. Only 36 patients were diagnosed with mediastinitis out of 1,542 sternotomies, with 20 in the VAC group and 16 in the conventional treatment group. The small sample size limits the study's statistical power and the ability to generalise the findings to a larger population. The second is the single-centre experience. The study was conducted in a single tertiary care centre. Practices, protocols, and patient populations vary across institutions, limiting the external validity and generalizability of the results. The third is the heterogeneous surgical procedures. The patients underwent various cardiac surgeries (e.g., CABG, valve replacement, congenital repairs). Different procedures come with varying levels of risk for infection and recovery, which may affect the study outcomes. Given the limitations of this retrospective, single-centre study, future research can be explored: prospective, multicentre studies. Larger, prospective studies across multiple centres would enhance the generalisability of findings and reduce biases inherent in retrospective designs. This would help validate the observed benefits of VAC therapy in broader patient populations. Moreover, through cost-benefit analysis, a detailed economic analysis comparing total treatment costs, including hospital stay, antibiotic use, surgical re-interventions, and outpatient care, would provide valuable insight into the financial viability of adopting VAC therapy as a standard approach.

## Conclusions

Mediastinitis is a life-threatening complication that can arise after cardiac surgeries performed through the median sternotomy approach. It is linked to higher rates of morbidity and mortality, reduced life expectancy, and adverse effects on the patient’s psychological well-being. Traditional treatment methods, such as wound debridement and irrigation, often result in higher mortality rates, increased chances of re-infection, and prolonged recovery periods. By contrast, VAC therapy offers significant advantages over conventional approaches. The salient points of VAC therapy include the following: (a) VAC/NPWT includes stabilises the wound, reduces oedema, reduces the bacterial load, improves tissue perfusion, and stimulates granulation tissue. It will improve the possibility of spontaneous wound healing and reduce the need for major plastic surgical procedures. VAC therapy is a simple and effective substitute for the management of various wounds compared to conventional dressings in terms of reduction in wound size, treatment duration, and cost. (b) VAC is a good alternative/adjunct to standard wound care, especially for difficult wounds. (c) It reduces the extent of reconstructive procedures. (d) The optimum pressure setting is 125 mm Hg. (e) Intermittent suction is better than continuous suction. (f) There are logistical benefits of VAC over conventional wound care methods. (g) The cost of VAC is comparable to standard wound care methods, and in the long term, it has cost benefits.
